# Impact of Vitamin D Status Correction on Serum Lipid Profile, Carboxypeptidase N and Nitric Oxide Levels in Saudi Adults

**DOI:** 10.3390/ijms24097711

**Published:** 2023-04-23

**Authors:** Sobhy M. Yakout, Saba Abdi, Alhanouf H. Alaskar, Malak Nawaz Khan Khattak, Abeer A. Al-Masri, Nasser M. Al-Daghri

**Affiliations:** 1 Department of Biochemistry, College of Science, King Saud University, Riyadh 11451, Saudi Arabia; syakout@ksu.edu.sa (S.M.Y.); sabdi@ksu.edu.sa (S.A.); hanouf.askar@gmail.com (A.H.A.); mkhattak@ksu.edu.sa (M.N.K.K.); 2 Department of Physiology, College Medicine, King Saud University, Riyadh 11451, Saudi Arabia; aelmasri@ksu.edu.sa

**Keywords:** 25(OH) D, vitamin D supplements, nitric oxide, carboxypeptidase N

## Abstract

This study aimed to determine the impact on the lipid profile, carboxypeptidase N (CPN) and nitric oxide (NOx) associated with vitamin D (VD) status correction among Saudi adults with VD deficiency. A total 111 VD deficient (25(OH)D < 50 nmol/L)) adult Saudis aged 18–50 years old (57 females and 54 males) were enrolled in this 6-month interventional study. They were given 50,000 IU VD weekly for the first 2 months and then twice a month for the next 2 months, followed by 1000 IU daily for the last 2 months. The fasting lipid profile and the blood glucose, VD, NOx and CPN concentrations were measured at baseline and after intervention. Post-supplementation, the median VD was significantly higher (*p* < 0.001) in females [58.3 (50.6–71.2)] and males [57.8 (51.0–71.8)]. HDL cholesterol significantly increased (*p* = 0.05) and NOx significantly decreased (*p* = 0.02) in males post-supplementation. Triglycerides were positively associated with NOx in all subjects before (r = 0.44, *p* = 0.01) and after (r = 0.37, *p* = 0.01) VD status correction. There was a significant increase in serum levels of CPN2 (*p* = 0.02) in all subjects. Furthermore, CPN was inversely correlated with NOx (r = −0.35, *p* = 0.05) in males post-supplementation. In conclusion, VD status correction reduced serum NOx, particularly in males. The inhibition of NOx synthesis may be responsible for the anti-inflammatory effects of VD supplementation. An inverse association was found between NOx and CPN2.

## 1. Introduction

The prevalence of vitamin D (VD) deficiency, defined as having a circulating 25 hydroxyvitamin (OH) D less than 50 nmol/L or 20 ng/mL, is now considered a major global health concern [1]. This is especially true in low- to middle-income countries where VD deficiency in adults ranges from 51–99% [2]. A study conducted among South Asian countries (Bangladesh, India, Pakistan, Nepal, Bhutan, Maldives, Sri Lanka, and Afghanistan) found a high prevalence of VD deficiency in the adult population [3]. Despite the abundance of sunshine, Saudi Arabia (SA) has also seen a high prevalence of VD deficiency across all ages and genders [4]. The 10-year (2008–2017) overall prevalence of VD deficiency for all age groups was 73.2% [5].

The importance of VD in human metabolism has been established due to its role in calcium and bone homeostasis [6]. Recent research has linked VD deficiency to a range of chronic diseases, including atherosclerosis, hypertension, diabetes, autoimmune diseases, cancer, cognition, and dental disorders [1,2,7,8,9,10,11,12]. It is, therefore, essential to understand which biochemical markers are affected by VD supplementation in order to effectively address VD deficiency.

Carboxypeptidase N (CPN) is a plasma metalloprotease with zinc metal that cleaves basic amino acids lysine or arginine from the C terminal of peptides and proteins [13]. CPN is important in the regulation of peptides (i.e., kinins and anaphylatoxins) and plays an important role in protecting the body from the excessive build-up of potentially deleterious peptides that normally act as local autocrine or paracrine hormones [14]. It is composed of two low macromolecular weight (50 KDa) CPN1 active subunits and two high molecular weight (83 kDa) CPN2 glycosylated subunits that keep the enzyme in circulation [13]. CPN has distinguishing features from other carboxypeptidases types since it is constitutively active and stable, having multiple subunits with higher molecular mass [15].

Human CPN was discovered in 1962 as an enzyme that inactivates bradykinin by cleaving its carboxy-terminal arginine. It was also referred to as kininase I [14]. In 1970, it was demonstrated that CPN inactivated the complement anaphylatoxins C3a, C4a, and C5a by the removal of their carboxy-terminal arginine [15]. Since then, other proteins have been proposed as substrates for CPN, including creatine kinase MM (CK-MM) [16]. So far, it is difficult to elucidate the role of CPN in disease processes. CPN is constitutively expressed in the liver and secreted into the bloodstream at approximately 30 μg/mL. It is an important enzyme that regulates biologically active peptides, including complement anaphylatoxins and kinins, by removing carboxy-terminal arginine or lysine. It is a member of a larger family of mammalian zinc carboxypeptidases. Furthermore, carboxypeptidases were involved in the digestion of food (pancreatic carboxypeptidases A1, A2, and B). However, most of the known carboxypeptidases are not involved in catabolism; they help to mature proteins (e.g., post-translational modification) or regulate biological processes. For example, the biosynthesis of neuroendocrine peptides such as insulin requires a carboxypeptidase. Carboxypeptidase also functions in blood clotting, wound healing, growth factor production, reproduction, and many other processes [17,18]. 

On the other hand, nitric oxide (NOx) is a pleiotropic signaling molecule produced by the endothelium and is present in almost every cell type [19]. NOx and its derivatives, nitrate and nitrite, have a variety of functions in human metabolism, including inflammation, oxidative stress, vasodilation, cardiac function, reproduction, gene transcription and translation, post-translational modifications [20,21,22,23], heart disorder, hypertension, and diabetes [24,25,26]. NO has three molecular mechanisms that mediate its biological activities. Firstly, it reacts with transition metals to regulate enzyme activity. Secondly, it induces the formation of S-nitrosothiols to modify protein activity. Thirdly, it reacts with superoxide anion to form peroxynitrite, which is a powerful oxidant that modifies proteins, lipids, and nucleic acids. The direct effects of NO predominate at low concentrations, while the indirect effects become more important at higher concentrations.

The active VD (1,25-dihydroxyvitamin D [1,25(OH)2D]) regulates the production of NOx and/or the expression of inducible NOS (iNOS) in different cells including endothelial cells, osteoblasts, microglial cells, macrophages, and astrocytes [27,28,29]. The relationship between VD and NOx has been extensively studied in cell lines and animal models and in both healthy individuals and patients [27,29,30,31]. Some studies have reported that VD is a direct transcriptional regulator of endothelial NOS and arterial stiffness [32]. The relation between CPN and NO is also well established, with CPN helping to increase NO production by increasing the release of arginine during inflammatory states [33]. Furthermore, CPN plays an important role in the regulation of inflammation by inactivating anaphylatoxins via cleaving arginine residue in the peptide. 

In the present study, the associations of CPN and NOx levels with serum 25(OH) D in response to VD supplementation in healthy adults of Saudi ethnicity were investigated. 

## 2. Results

**Figure 1:**
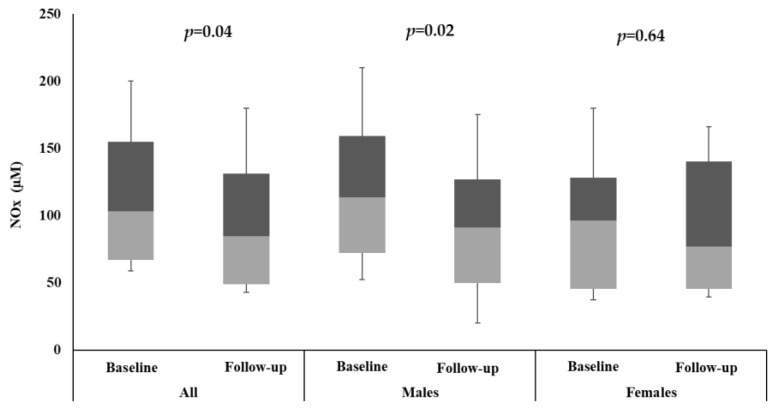
Median change in NOx (µM) at baseline and follow-up.

A total of 111 VD deficient (25(OH)D < 50 nmol/L) adult Saudis aged 18–50 years old (57 females and 54 males) were enrolled in this study (Table 1). HDL cholesterol, CPN (*p* = 0.02), and high VD (*p* < 0.001) were found to be increased, whereas NOx was reduced (*p* = 0.04) in all subjects (Figure 1). No significant differences were noted between serum levels of glucose, total cholesterol, LDL cholesterol, and triglycerides post-supplementation. Males had higher HDL cholesterol while females had a modest but non-significant increase in CPN2. The serum levels of NOx were significantly reduced in the male group but not in the female group. At baseline, NOx was significantly higher in males than females.

Table 2 showed the bivariate associations between NOx with other parameters. There was a significant positive correlation between NOx and glucose among all group at follow-up. Serum triglycerides were significantly and positively correlated to NOx both at baseline and follow-up in all subjects, particularly in females. Both VD and CPN2 were inversely correlated with NOx. A significant inverse correlation was also noted between CPN2 and NOx in males post-VD supplementation.

**Figure 2:**
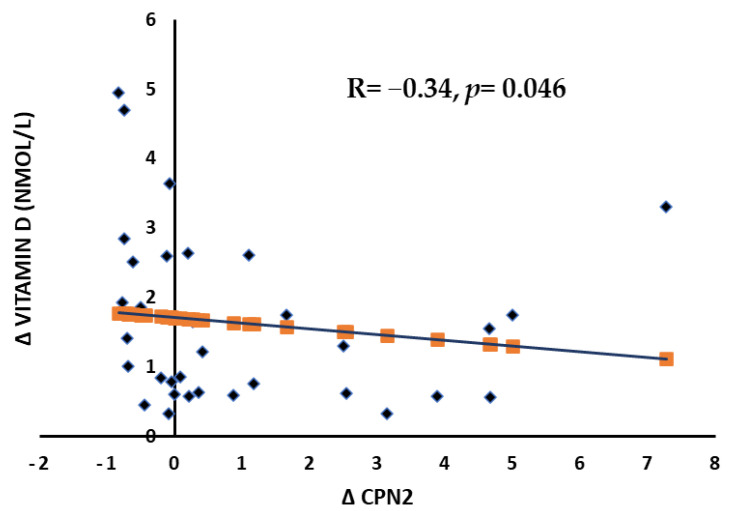
Inverse association between VD and CPN delta changes in female subjects.

The bivariate associations of delta (Δ) changes in the correlation matrix for CPN2 and Nox with other parameters are shown in Table 3. No significant associations were observed in the glucose, cholesterol profile, and triglycerides. Delta changes in 25(OH)D with CPN2 showed a significant inverse association (R = −0.34, *p* = 0.046) in the female group (Figure 2). Finally, in the CPN2, delta changes in NOx showed a significant positive association (*p* < 0.001).

## 3. Discussion

VD is a multifunctional hormone with an important role in the human body. Some evidence suggests that VD deficiency is associated with an increased risk of cardiovascular diseases, type 2 diabetes, multiple sclerosis, and some types of cancer. Some previous studies on the association of VD with NOx have been contradictory. In line with this study, Al-Daghri et al. [34] reported that serum 25(OH)D and NOx showed a significant inverse association in adolescent Saudi subjects, more so in males than in females [34]. A study conducted on rat lungs treated with lipopolysaccharides so as to induce iNOS expression noted that, during inflammation, a carboxypeptidase substrate, after cleavage by cellular carboxypeptidase, can provide sufficient arginine, thereby, stimulating NO generation [33]. Another study conducted on macrophage cell line RAW 264.7 supports the hypothesis that free arginine is increased by the action of carboxypeptidase (D) on numerous pro-inflammatory mediators, resulting in an increase in free arginine, followed by an increase in NOx levels [34]. 

NOx is a reactive radical that regulates cellular functions in both physiological and pathological conditions [35]. NOx has a variety of activities in the cardiovascular system, including the regulation of vascular tone and endothelial barrier function [36]. In physiological states, NOx can serve a protective function, but under conditions of high output, it may contribute to tissue damage by reacting with superoxide to form peroxynitrite, a strong oxidant [37]. Several disorders are caused by high levels of NOx, such as autism spectrum disorder, arthritis, and lung disease [38]. Therefore, NOx inhibition may help to manage these disorders. In addition to participating in the inflammatory response via its physiological effects, NOx also has the ability to regulate the expression of inflammatory proteins [39] via its regulation of the transcription factor, nuclear factor-kB [40]. Thus, it has been suggested that NOx may exert both deleterious and protective effects in inflammatory conditions, such as sepsis, depending on species, timing, the cell type, the inflammatory stimulus, the NOx concentration, and the NOx-related metabolites generated [41]. NOx production, by the inducible isoform of NOx synthase (iNOS), plays a pivotal role in numerous diverse pathophysiological processes. Inducible NOS is expressed in many cell types in response to a diverse range of inflammatory cytokines, including interleukins (IL)-1b, IL-2, interferons (IFN), tumor necrosis factor (TNF), and lipopolysaccharides [42].

Because the subjects of the present study were overweight, with prediabetes as well as VD deficiency, we assume they had an underlying subclinical inflammatory condition that could have caused enhanced NOx synthesis. Thus, the reduction in their serum NOx levels after VD supplementation may have been due to reduced inflammation.

Obesity may interfere with the maintenance of an optimal state of health. The excess of macronutrients in the adipose tissues stimulates them to release inflammatory mediators such as TNF and interleukin-6 (IL-6) that stimulate the liver to synthesize and secrete C-reactive protein (CRP). As a risk factor, inflammation is an embedded mechanism for the development of cardiovascular diseases including coagulation, atherosclerosis, metabolic syndrome, insulin resistance, and diabetes mellitus [43]. Elevated concentrations of CRP, IL-6, and TNF with suboptimal VD levels have been associated with an increased risk of cardiovascular events, glucose metabolism disorders, neurodegenerative diseases, and overall mortality [44].

VD supplementation is considered one of the most effective ways to improve the lipid profile. One prospective study found that VD supplementation over 12 months increased HDL cholesterol levels among adults, consequently reducing the atherosclerotic cardiovascular disease risk score [45]. Furthermore, a consistently positive correlation between VD status and HDL-C is in accordance with several observational studies conducted with children [46].

In the present study, HDL cholesterol increased overtime for both groups, with a significant increase in males and borderline significance in females. This finding supports many hypotheses that VD will increase or regulate good cholesterol, especially in males, who are at higher risk for CVD and heart disease. We have previously observed that VD status correction among VD deficient Saudi adults corresponds to a substantial reduction in atherosclerotic cardiovascular disease [45] since they do not have a high level of estrogen, as women do. Higher levels of estrogen in premenopausal women have been found to increase levels of HDL; thus, women are much less susceptible to coronary heart disease (CHD) and other atherosclerotic diseases compared to men [46,47].

NOx levels were significantly higher in males than in females, significantly at baseline, and triglycerides were significantly positively related to NOx in all subjects; this is in line with our previous study [48]. Moreover, one study hypothesized that nitric oxide (NO) production is associated with serum lipid concentrations and body mass index (BMI), and these associations are stronger in males than in females [49]. Post-VD supplementation, NOx levels were significantly reduced in males but not in females. This indicates that VD supplementation can influence NOx production. Thus, the results of the present study support the extraskeletal role of VD in regulating endothelial homeostasis via the regulation of NOx production, mainly in males. However, NOx is synthesized from L-arginine via nitric oxide synthase’s (NOS) activity.

CPN is a very important part of the body’s defense against the build-up of potentially harmful peptides that normally act as autocrine or paracrine hormones in the area. CPN is different from other carboxypeptidases in the same family because it is always active and stays stable in the bloodstream. It is also the only carboxypeptidase in this family with more than one subunit and a much higher molecular mass. However, questions about the relationship between VD and CPN have yet to be answered. The present study measured levels of CPN and found a significant increase in CPN post-VD supplementation in the overall population. Furthermore, the associated study also showed that CPN was significantly inversely correlated to NOx in males after VD supplementation.

In the present study, CPN was not directly correlated to NOx; thus, the present study does not support the view that CPN increases NOx production. Previous studies have suggested that CPN can enhance NOx production, mainly in inflammatory conditions. In contrast, some researchers oppose this hypothesis, and state that the effect on iNOS in the blood is limited, so it may be unlikely that CPN will affect NOx production appreciably in the circulation. For example, even after massive complement activation, there may be only as much as 10 μM of free arginine generated from the action of CPN on the complement anaphylatoxins C3a, C4a, and C5a, and this would only increase the plasma concentration of arginine by 10% at most and, as discussed above, would not affect the production of NO via constitutive NOS. Support for CPN not affecting NO production in the circulation was recently shown in C3 and C3a receptor-deficient mice [50]. These mice, when subjected to an LPS challenge, did not show any change in NO production in their circulation compared to the wild type controls that were similarly treated.

In the present study, VD supplementation increased CPN levels, but the increase in CPN did not increase NOx production. This could be because VD caused a reduction in inflammatory conditions. VD may reduce inflammation by increasing CPN, which has been reported to reduce inflammation by inactivating bradykinin and complements.

Bradykinin is a naturally occurring neuropeptide (plasma protein). It is a pharmacologically active kinin, which is considered either a cardioprotective or proinflammatory agent [51]. It is very similar to kallidin, which has the same sequence but with an additional N terminal lysine. They appear to be important regulators of cardiovascular function, being increasingly noted as likely participants in the actions of drugs that affect the heart, kidney, and circulation. It has been shown that bradykinin acts via two receptors, B1 and B2, that differ in the mechanism by which they are regulated. Both are typical G-protein-coupled receptors [52], which have very similar effects. The roles of B1-receptors in inflammation are in leucocyte recruitment, the initiation of inflammatory responses, and the physiology of pain. It has been implicated in several pathophysiological conditions such as hypertension, diabetes, and other cardiovascular and renal disorders. Although bradykinin has multiple beneficial actions, some undesirable effects have been reported, such as, oedema, and angioedema, which can be reversed by bradykinin antagonists. CPN2 levels will be reduced because of its function to inactivate bradykinin by cleaving its carboxy-terminal arginine and to inactivate the complement anaphylatoxins C3a, C4a, and C5a by removing their carboxy-terminal arginine. In this situation, bradykinin levels will be induced, acting as a pro-inflammatory agent. Bradykinin will acutely increase the levels of nitric oxide (NO) production by activating endothelial NO synthase (eNOS), and this increase is in part correlated with the enhanced phosphorylation/dephosphorylation of eNOS by several protein kinases and phosphatases [53].

The authors acknowledge several limitations. The lack of a control group gives it the principal disadvantage of having potential biases from confounding factors. We do acknowledge that the presence of a control arm would have made the study much more scientifically sound. Another limitation is the lack of baseline information affecting VD statuses, such as dietary VD and sun exposure, which can improve the VD status of participants. Additionally, body composition calculations using BMI and WHR were not recorded at the end of the study, which could explain the differences in results according to sex. Lastly, while the sample size was sufficient to elicit the required significance, a larger sample size might have strengthened the evidence on the influence of VD in the general population. Despite the limitations, the study is well supported and the first in this homogenous ethnic group to observe the modest benefits of VD correction in terms of reducing NOx and HDL cholesterol levels and increasing CPN2 levels among Arab adults with VD deficiency. This may give new insight for scientific and medical fields.

## 4. Materials and Methods

**Figure 3:**
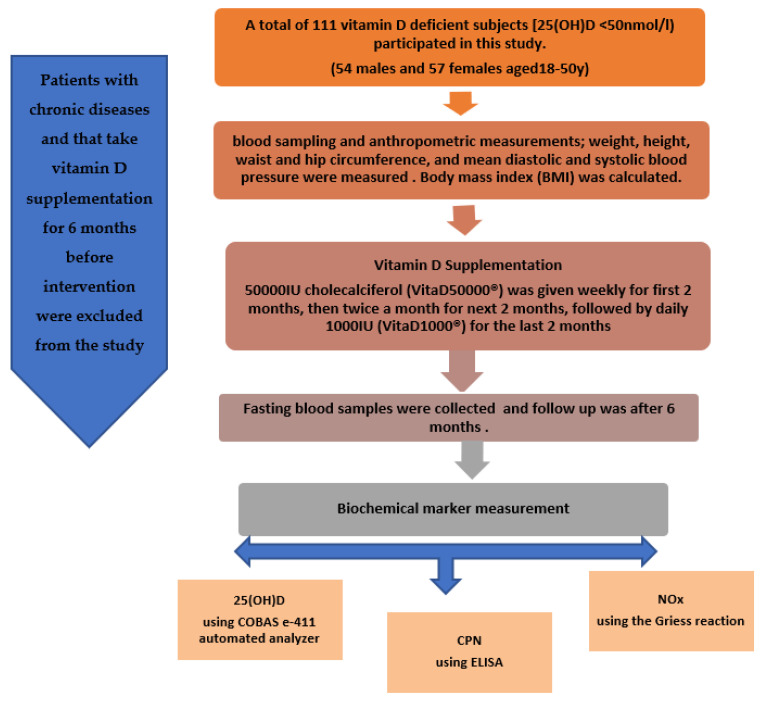
Flow chart of the study participants describing their participation and the exclusion criteria used in this study.

A flow chart describing the study population is provided in Figure 3, showing the total number of subjects (N = 111) selected from Riyadh and the exclusion criteria used in this study.

### 4.1. Study Design and Population

A total of 111 VD deficient subjects [25(OH)D nmol/L], aged 40.4 ± 11.4 years (54 males and 57 females with an age range of 18–50 years), were admitted to the study to allow dosing in all periods. VD deficiency was defined as 25(OH)D < 50 nmol/L, sufficiency as 25(OH)D ≥ 50 nmol/L, and adequacy as 25(OH)D > 75 nmol/L [47]. Individuals with serious clinical conditions such as anemia, type 2 diabetes mellitus (T2DM), cancer, cardiovascular disease, liver dysfunction, renal dysfunction, or thyroid dysfunction have been excluded from the study. Subjects taking VD supplementation for 6 months before the intervention were excluded at the screening phase. Ethics approval (No. FWA00018774) was obtained from the ethics committee of the College of Medicine Research Center, King Saud University in Riyadh, KSA. After their agreements, all patients who participated in the study signed written consent. All the individuals were asked to visit primary health care centers for blood sampling and anthropometric measurements, including weight, height, waist and hip circumference, and mean diastolic and systolic blood pressure. Body mass index (BMI) was calculated by dividing weight (kilograms) by height (square meters), and individuals with a BMI ≥ 25 kg/m^2^ and ≥30 kg/m^2^ were considered overweight and obese, respectively. About 10 mL of venous blood samples were collected from each individual and centrifuged for separation of serum samples. The remaining blood and serum samples were immediately delivered to the Chair for Biomarkers of Chronic Diseases (CBCD) in King Saud University, Riyadh, KSA, in specialized containers for biochemical analyses and stored at −80 °C.

### 4.2. VD Intervention

VD supplementation was carried out in accordance with the previously published study on local recommendations for VD status correction [47]. A dose of 50,000 IU cholecalciferol (VitaD50000^®^, Synergy Pharma, Dubai, UAE) was given weekly for the first 2 months and then twice a month for the next 2 months, followed by 1000 IU (VitaD1000^®^, Synergy Pharma, Dubai, UAE) daily for the last 2 months. To ensure compliance, patients were asked to return unused capsules at every follow-up visit before being given another set of supplements. Subjects with non-compliance were excluded from the study. Patients were also regularly encouraged via short message service (SMS) to take the recommended VD dose. The orientation and intervention were performed by a qualified nutritionist, physician, and nurses in the health care center, and all the procedures followed the ethical principles advised in the Helsinki Declaration. The intervention study was approved by the Ethics Committee of the College of Science, King Saud University (KSU), Riyadh. Blood samples (5 mL) were obtained at baseline and after 6 months to monitor the achievement of full VD status correction.

### 4.3. Measurement of Biochemical Markers

Serum 25(OH) VD was analyzed using the COBAS e-411 automated analyzer (Roche Diagnostics, Indianapolis, IN, USA). All experiments were performed in triplicate, and control experiments were performed to assure the quality of reproducible results. The inter- and intra-assays were applied for estimation of serum 25(OH)D. Coefficients of variation (CV) were taken as 8.0 and 5.6%, with a lower detection limit (LOD) of 7.5 nmol/mL [19]. The fasting lipid profile and blood glucose were determined using a chemical analyzer (Konelab 20XTi, Termo Electron Corporation, Vantaa, Finland) [54]. The measurement of nitrate/nitrite concentration or of total nitrate and nitrite concentration was routinely used as an index of NOx production [15]. The concentration of serum NOx was measured using the Griess reaction [20]. Serum NOx concentration was determined from the linear standard curve established using 0–50 µM sodium nitrate. The inter- and intra-assay coefficients of variation were set at 5.2% and 4.4%, respectively, while the recovery of the assay was 93 ± 1.5%. Determination of CPN was carried out using human CPN ELISA Assay Kit, according to manufacturer’s instructions. (LifeSpan BioSciences, Inc., Seattle, WA, USA). 

### 4.4. Sample Size Calculation and Statistical Analysis

Sample size calculation was carried out based on data obtained from previous findings ascertaining the effects of 25(OH)D correction [45,55]. G*Power was used to determine sample size given the effect size of 0.27 and the power of 0.85. The minimum required total sample is N = 100 given α = 0.05.

Data were analyzed using SPSS (version 21, IBM). Continuous data were presented as mean ± standard deviation (SD) for Gaussian variables, and non-Gaussian variables were presented in median (1st and 3rd) percentiles. All continuous variables were checked for normality using the Kolmogorov–Smirnov test; if not normal, the non-Gaussian variable was transformed to the log transform. Categorical variables were presented in percentages (%). An independent pair t-test was used to check the mean and median changes of the pre- and post-Gaussian variables, and Wilcoxon tests were used for non-Gaussian variables even if they transform; however, as stated, this is not normal for gaussian variables. Correlations between variables were conducted using Spearman’s and Pearson’s correlation analysis. A *p*-value < 0.05 was considered statistically significant.

## 5. Conclusions

In conclusion, the present study suggests that VD supplementation can influence CPN, NOx, and HDL cholesterol production, particularly in males. VD supplementation increased CPN levels but decreased HDL cholesterol and NOx production, possibly due to the reduction in inflammatory conditions caused by VD. CPN was significantly inversely correlated with NOx among male subjects’ post-supplementation. Further research is needed to fully understand the mechanisms underlying these effects and to determine the optimal dosages for VD supplementation.

## Figures and Tables

**Table 1:** Clinical characteristics of the subjects before and after 6 months based on subject responses.

		All			Males			Females	
	Baseline	Follow-Up	Mean Change	Baseline	Follow-Up	Mean Change	Baseline	Follow-Up	Mean Change
N (M/F)	111 (54/57)			54			57		
Age (year)	39.5 ± 9.4			42.2 ± 8.4			36.9 ± 9.7		
BMI (kg/m^2^)	29.3 ± 4.8			28.96 ± 3.8			29.6 ± 5.7		
WHR	0.93 ± 0.10			0.99 ± 0.06			0.87 ± 0.10		
Systolic BP (mmHg)	125.5 ± 13.7			132.4 ± 11.8			117.3 ± 11.1		
Diastolic BP (mmHg)	79.6 ± 9.7			82.8 ± 7.7			75.9 ± 10.5		
Glucose (mmol/L)	5.62 ± 1.1	5.53 ± 0.89	−0.09 (0.11)	5.75 ± 1.1	5.85 ± 0.9	0.10 (0.2)	5.50 ± 1.1	5.23 ± 0.8	0.27 (0.2)
Total Cholesterol (mmol/L)	5.18 ± 1.2	5.19 ± 1.3	0.02 (0.10)	5.13 ± 1.1	5.19 ± 1.2	0.07 (0.14)	5.22 ± 1.3	5.20 ± 1.3	−0.03 (0.15)
HDL Cholesterol (mmol/L)	1.04 ± 0.4	1.15 ± 0.4 *	0.12 (0.05)	0.98 ± 0.3	1.1 ± 0.3 *	0.08 (0.04)	1.09 ± 0.5	1.25 ± 0.5	0.16 (0.1)
LDL Cholesterol (mmol/L)	3.29 ± 1.0	3.21 ± 1.1	−0.08 (0.10)	3.2 ± 0.9	3.2 ± 0.1.1	0.004 (0.1)	3.37 ± 1.1	3.21 ± 1.0	−0.16 (0.1)
Triglycerides (mmol/L)	1.4 (0.9–2.1)	1.5 (1.0–2.1)	0.01 (0.09)	1.5 (1.0–2.3)	1.6 (1.1–2.2)	0.06 (0.13)	1.4 (0.9–2.2)	1.3 (0.9–2.0)	−0.04 (0.13)
25(OH)D (nmol/L)	31.9 (22–39)	58.1 (51–71) **	31.4 (1.7)	32.7 (24–42)	58 (51–71) **	30.1 (2.5)	31.3 (20–38)	57.8 (51–72) **	32.5 (2.3)
CPN (ng/mL)	299 (152–522)	432(185–744) *	168 (57.5)	330(183–525)	475 (225–681)	148.8 (78.1)	221 (100–528)	424 (139–910)	190.2(86.1)
NOx (µM)	103.2 (67–155)	84.5 (49–131) *	−16.7 (6.9)	113.5 (72–159)	91.1 (50–127) *	−25.9 (9.9)	96.2 (46–128)	77 (46–140)	−7.45 (9.4)

Note: Data presented as mean ± SD and median (1st–3rd) percentiles for Gaussian and non-Gaussian variables. * and ** represent *p*-values that are significant at 0.05 and 0.01, respectively, for paired *t*-test.

**Table 2:** Association of log NOx with measured parameters.

Parameters	All		Males		Females	
	Baseline	Follow-Up	Baseline	Follow-Up	Baseline	Follow-Up
Glucose (mmol/L)	0.03	0.22 *	0.07	0.11	0.10	0.25
Total Cholesterol (mmol/L)	0.27 **	0.07	0.33 *	0.16	0.26	0.02
HDL Cholesterol (mmol/L)	−0.11	−0.09	0.36 *	0.19	−0.25	−0.21
LDL Cholesterol (mmol/L)	0.12	0.02	0.07	0.13	0.14	−0.08
Log Triglycerides	0.44 **	0.37 **	0.46 **	0.23	0.42 **	0.45 **
Log 25(OH) D	−0.10	−0.04	−0.01	0.07	−0.26	−0.03
Log CPN	−0.08	−0.16	−0.06	−0.35 *	−0.13	−0.06

Note: Data presented as co-efficient of determination (R). * and ** represent *p*-values that are significant at 0.05 and 0.01, respectively.

**Table 3:** Correlation matrix for CPN2 and NOx (delta change) with other parameters.

Parameters	Δ CPN2			Δ NOx		
	All	Males	Females	All	Males	Females
Δ Glucose	0.2	0.23	0.24	0.11	0.17	0.12
Δ Total Cholesterol	0.11	0.04	0.15	0.12	−0.02	0.17
Δ HDL Cholesterol	−0.1	−0.19	0.03	−0.12	−0.24	−0.03
Δ LDL Cholesterol	0.16	0.11	0.14	0.13	0.04	0.14
Δ Triglycerides	−0.08	−0.21	0.16	−0.04	−0.1	0.1
Δ 25(OH)D (nmol/l)	−0.17	−0.03	−0.34 *	−0.12	−0.03	−0.27
Δ CPN	1	1	1	0.92 **	0.93 **	0.92 **
Δ NOx	0.92 **	0.90 **	0.90 **	1	1	1

Note: Data presented as co-efficient of determination (R). * and ** represent *p*-values that are significant at 0.05 and 0.01, respectively.

## Data Availability

Data are available upon request from the corresponding author.
